# Cross-reactions of sera from dogs infected with *Angiostrongylus vasorum* in commercially available *Dirofilaria immitis* test kits

**DOI:** 10.1186/1756-3305-5-258

**Published:** 2012-11-13

**Authors:** Manuela Schnyder, Peter Deplazes

**Affiliations:** 1 Institute of Parasitology, Vetsuisse Faculty, Winterthurerstrasse 266a, 8057, Zürich, Switzerland

**Keywords:** *Angiostrongylus vasorum*, *Dirofilaria immitis*, Antigen detection, Cross-reactions, Dogs

## Abstract

**Background:**

*Dirofilaria immitis* and *Angiostrongylus vasorum* are both important potentially fatal canine nematodes with overlapping endemic areas, especially in Europe. The preadult and adult stages of both species are living in the Arteria pulmonalis and the right heart, and diagnostically detectable circulating parasite antigens have been demonstrated for both species. For the detection of *D. immitis* infections, a variety of commercial tests have been developed, however, they have not been evaluated for cross-reactions against circulating antigens of *A. vasorum.*

**Methods:**

In this study, potential cross-reactions of sera from 16 dogs, which were experimentally infected with *A. vasorum* and which had circulating antigens as confirmed by a species-specific ELISA, were evaluated for the detection of *A. vasorum* antigen in six commercially available *D. immitis* test kits.

**Results:**

In three fast tests (Witness® Dirofilaria, SensPERT® Canine Heartworm, SNAP® 4Dx® Plus), all sera were negative. One fast membrane ELISA (SNAP® HTWM RT Test) was positive with four sera (25%), and one serum delivered a non-valid result twice. In the PetChek® HTWM PF Test, depending on the interpretation protocol, 5 or 8 dogs (31.2 – 50%) were positive. With the DiroCHEK®-ELISA, a single *A. vasorum*-infected dog (6.2%) tested positive.

**Conclusions:**

Due to potential cross-reactions with *A. vasorum* in commercially available test kits for the detection of *D. immitis* antigen, the simultaneous use of highly specific diagnostic methods for the differentiation of these two canine heart worms is recommended.

## Background

The adult stages of *Dirofilaria immitis*, a filarial nematode, and *Angiostrongylus vasorum*, a metastrongylid nematode, are both localized in the Arteria pulmonalis and the right heart of their definitive hosts. Dogs, foxes and some other carnivores are the definitive hosts of both parasites, while Culicidae and Gastropoda are the intermediate hosts of *D. immitis* and *A. vasorum*, respectively.

In Europe, *D. immitis* is present in coastal Mediterranean areas with expansion northwards, while in North America the parasite has expanded from the south-eastern coastal areas northwards and westwards [1] up to Canada [2]. *A. vasorum* was diagnosed for the first time in France in 1913 [3], but it is only recently that this parasite has regained attention within the veterinary community [4,5]. Its presence has been increasingly reported from several new areas in and outside Europe (reviewed in [6]). Reports of an increasing number of cases of canine angiostrongylosis, as well as the development of new diagnostic tools such as ELISAs [7-10] or biomolecular techniques [11,12] may have contributed and also incentivised epidemiological studies, confirming the presence of this parasite in dogs, foxes and snails throughout Europe. The Atlantic provinces of Newfoundland and Labrador are the only regions actually affected by *A. vasorum* in North America, with a potential for expansion to further regions [4,13,14]. Overlapping areas in large parts of southern Europe with the presence of both *A. vasorum* and *D. immitis* have therefore to be accounted for. Furthermore, in non-endemic areas of *D. immitis*, this agent has to be considered based on anamnestic information (travelling with pet dogs or imports) and differentiated from *A. vasorum* infections.

Fatal clinical consequences of *D. immitis* infection are usually prevented by the monthly use of macrocyclic lactones in known endemic areas [15-17], and treatment of dirofilariosis is based on the intramuscular application of the arsenic derivate melarsomine [18] and/or, alternatively, by eliminating the endosymbiont *Wolbachia* with doxycycline supported by administration of macrocyclic lactones [19]. Dogs infected with *A. vasorum*, instead, are treated using macrocyclic lactones such as moxidectin [20] or milbemycin-oxime [13], or applying fenbendazole [21]. Prophylactic treatment (with macrocyclic lactones) against potentially fatal canine angiostrongylosis is, as for dirofilariosis, recommended in highly endemic and well known areas [20].

The currently used diagnostic laboratory methods for the detection of these parasites are divergent. The diagnosis of *D. immitis* is based on the detection of microfilariae or circulating antigens released by mature adult female worms into the blood circulation, both being detectable starting from 6 months after infection [18]. A variety of tests have been developed for the detection of circulating antigens, employing lateral flow immunochromatographic techniques, membrane ELISAs or conventional ELISAs [22-24]. Test evaluations showed that the sensitivity of heartworm antigen tests depends on the worm burden, and the sex and age of the parasites [24-28], while the specificity of the kits is regularly indicated to be very high, between 95% and 100% [23,25,26,29,30]. However, only occasionally were potential cross-reactions evaluated in animals with natural or experimental infections with other helminths, mainly against other filarial nematodes such as *Dipetalonema reconditum*[22,31] or *Dirofilaria repens*, indicating that modern test kits may overcome cross-reactions detected in previously developed test kits for these parasites [32,33], and, rarely, against intestinal parasites such as *Ancylostoma caninum* and *Trichuris* spp. [22,34]. The most current diagnostic method for detection of *A. vasorum* infections in dogs is the isolation of first stage larvae (L1) from faecal samples, which are produced by the parasites approximately 6–7 weeks after infection. Larval migration techniques such as the Baermann-Wetzel method [35] are commonly adopted. Furthermore, ELISAs for the detection of antibodies against *A. vasorum* have been described [7,9], and, recently, tests for the detection of circulating antigen of *A. vasorum* have been developed. These latter ones have been evaluated for cross-reactions against *Crenosoma vulpis*[8,10] and also against intestinal parasites (*Toxocara canis, Ancylostoma caninum*) and, importantly, against *D. immitis*[8], showing a high specificity (94-100%).

Due to their common localization within the definitive hosts, their considerable size and particularly the well documented production of circulating antigens [31,36], it was argued that antigens of *A. vasorum* and *D. immitis* may share epitopes responsible for potential cross-reactions in antigen detection tests. This hypothesis has been confirmed during the development of the ELISA for the detection of circulating antigen for *A. vasorum*[8].

The aim of the study was to evaluate potential cross-reactions of sera from dogs experimentally infected with *A. vasorum* in six different commercially available tests for the detection of *D. immitis* antigen.

## Methods

A total of 16 sera from dogs experimentally inoculated with 200 third stage larvae (L3) of *A. vasorum* were obtained during previously performed studies [20,37]. Infection of dogs was confirmed by positive Baermann-Wetzel analyses [35], by the detection of circulating *A. vasorum* antigen [8] and by the presence of adult worms at necropsy adopting an established method of reverse lung perfusion [20]. The day of sample collection (between 55 and 356, mean 101) after inoculation (dpi) and the number of detected parasites at necropsy are shown in Table 1. Worm burdens varied between 10 and 170 adult parasites, with a mean of 66 worms per dog. *Dirofilaria immitis* infection was excluded based on the fact that the dogs were living in a non-endemic area under controlled experimental conditions, and at necropsy.

**Table 1:** **Comparative results of 16 sera from dogs experimentally inoculated with *Angiostrongylus vasorum *(Av) tested with 6 different diagnostic kits for the detection of *D. immitis *antigen and with an ELISA for detection of *A. vasorum *circulating antigen**[8]

**Dog-ID**	**Days post inoculation (dpi)**	**Worm burden (n)**	***A. vasorum *antigen detection (optical density)**^**1**^	**Diagnostic test kits for the detection of *Dirofilaria immitis *antigen**						
				**Witness®**	**SensPERT®**	**SNAP® HTWM RT**^**2**^	**SNAP® 4Dx® Plus**	**PetChek® (veterinary practice conditions)**^**3**^	**PetChek® (laboratory conditions)**^**3**^	**DiroCHEK ®**
Av 1	55	49	1.484	neg.	neg.	neg.	neg.	neg.	neg.	neg.
Av 2	55	54	1.825	neg.	neg.	neg.	neg.	neg.	neg.	neg.
Av 3	55	106	1.743	neg.	neg.	neg.	neg.	neg.	neg.	neg.
Av 4	55	129	1.400	neg.	neg.	neg.	neg.	neg.	neg.	neg.
Av 5	55	134	1.485	neg.	neg.	neg.	neg.	neg.	pos.	neg.
Av 6	59	57	0.567	neg.	neg.	neg.	neg.	neg.	neg.	neg.
Av 7	59	98	0.657	neg.	neg.	neg.	neg.	neg.	neg.	neg.
Av 8	76	32	0.263	neg.	neg.	not valid	neg.	low pos.	pos.	pos.
Av 9	76	42	0.520	neg.	neg.	neg.	neg.	neg.	neg.	neg.
Av 10	76	68	1.007	neg.	neg.	neg.	neg.	neg.	pos.	neg.
Av 11	90	13	0.879	neg.	neg.	neg.	neg.	neg.	pos.	neg.
Av 12	90	30	1.069	neg.	neg.	neg.	neg.	neg.	neg.	neg.
Av 13^4^	91	10	1.264	neg.	neg.	low pos.	neg.	low pos.	pos.	neg.
Av 14^4^	91	170	1.396	neg.	neg.	low pos.	neg.	low pos.	pos.	neg.
Av 15	286	36	2.007	neg.	neg.	low pos.	neg.	pos.	pos.	neg.
Av 16	356	24	1.357	neg.	neg.	low pos.	neg.	pos.	pos.	neg.

^1^: optical density values read at 405 nm.

^2^: SNAP® HTWM RT test differentiates between low positive (low pos.) and positive results.

^3^: The interpretation of PetChek® results can be done under an “In-clinic”-protocol based on subjective colour evaluation or under the “Laboratory protocol” by measuring the optical densities at 650 nm and a cut-off calculation based on positive and negative controls.

^4^: dog Av 13 and Av 14 were inoculated with 50 and 500 L3, respectively.

neg.: negative.

pos.: positive.

All tests were performed blinded with an identity code from 1 to 16, by veterinarians (fast tests) or by experienced laboratory technicians from the IPZ (ELISAs).

All sera were non-haemolytic, stored at −20°C and tested within 11–40 months after collection. The following test kits were used, adopting the manufacturer’s instruction and within the indicated expiry dates:

1) Witness® Dirofilaria, lateral flow (Synbiotics, San Diego, USA)

2) SensPERT® Canine Heartworm, lateral flow (VetAll Laboratories, Kyunggi-Do, South Korea)

3) SNAP® HTWM RT, membrane ELISA (IDEXX Laboratories, Westbrook, USA)

4) SNAP® 4Dx® Plus, membrane ELISA (IDEXX Laboratories, Westbrook, USA)

5) Petchek® HTWM PF Antigen Test , ELISA (IDEXX, Westbrook, USA)

6) DiroCHEK®, ELISA (Synbiotics San Diego, USA)

## Results

Single test results are shown in Table 1. All tests, with one exception (see Tab. 1, dog Av 8) fulfilled the criteria for test validity based on the results of positive and/or negative controls.

The ELISA for the detection of circulating *A. vasorum* antigen was highly positive for all experimentally infected dogs, with absorbance values (optical density read at 405 nm, OD) varying between 0.263 and 2.007 (cut-off value: 0.159, as previously described [8]), with a mean of 1.096.

In three fast tests (Witness®, SensPERT®, SNAP® 4Dx® Plus) all sera resulted negative, while in one fast membrane ELISA (SNAP® HTWM RT) four *A. vasorum* infected dogs were positive for *D. immitis* antigen, and one serum delivered a non-valid result twice. In the PetChek®-ELISA two methods for interpretation were adopted: following the instruction for veterinary practitioners based on eye detection, 5 dogs resulted positive for *D. immitis* infection, while following the instructions under laboratory conditions with OD measurements, a total of 8 dogs were seropositive. With the DiroCHEK®-ELISA, a single *A. vasorum* infected dog was *D. immitis* seropositive. With one exception (76 dpi), all cross-reactions were observed in dogs infected with *A. vasorum* for more than 90 days, with worm burdens varying from 10–170.

## Discussion

This study provides evidence of false positive reactions in *D. immitis* antigen detection kits with sera of dogs infected with *A. vasorum*. The two ELISAs for *D. immitis* detection (PetChek® and DiroCHEK®) and the membrane ELISA SNAP® HTWM RT showed single cross-reactions against *A. vasorum*, which had not been considered so far. In contrast, the adopted ELISA for the detection of circulating *A. vasorum* antigen has been developed evaluating different monoclonal antibodies, which had been selectively chosen based on their absence of cross-reactivity against *D. immitis* circulating antigens, resulting in an overall high specificity [8].

Generally, *D. immitis* antigen tests are considered to be more sensitive than microfilariae concentration methods or other procedures [38]. In particular, the ELISA technology has been shown to be more sensitive than lateral flow immunochromatography [26] for the diagnosis of heartworm infected dogs. Reasons for the occurrence of false negative results with sera of *D. immitis* – positive dogs have been discussed in previously performed studies evaluating different *D. immitis* test kits. Low worm burden and low number of female worms have been shown to reduce sensitivity of the tests [25,26,39]. However, increased sensitivity may be coupled with lower specificity and, importantly, with potential cross-reactions against *A. vasorum*. An unknown number of dogs with travel anamnesis and testing positive for circulating heartworm antigen may have falsely been diagnosed positive due to *A. vasorum* cross-reactions, and erroneously treated with melarsomine and/or macrocyclic lactones. Therefore, serological results for *D. immitis* should be confirmed or excluded by additional diagnostic tests (Knott’s test for microfilariae of *D. immitis,* or serology or Baermann migration test for L1 of *A. vasorum*) or diagnostic imaging frequently delivering pathognomonic findings for heart dirofilariosis [40,41] or angiostrongylosis [42,43].

## Conclusions

In this study we confirmed that sera of dogs infected with *A. vasorum* cross-react in commercially available test kits for the detection of circulating *D. immitis* antigen. The simultaneous use of highly specific diagnostic tools is recommended for epidemiological studies where both heart worm species occur or for individual dogs with a suspected heart worm infection.

## Competing interests

The authors declare that they have no competing interests.

## Authors’ contributions

MS participated in the design of the study, collected the samples, carried out the diagnostic assays and drafted the manuscript. PD conceived the study and implemented the draft of the manuscript. Both authors have read and approved the final manuscript.
